# Decoding Kinematic Information From Primary Motor Cortex Ensemble Activities Using a Deep Canonical Correlation Analysis

**DOI:** 10.3389/fnins.2020.509364

**Published:** 2020-10-16

**Authors:** Min-Ki Kim, Jeong-Woo Sohn, Sung-Phil Kim

**Affiliations:** ^1^Department of Biomedical Engineering, Ulsan National Institute of Science and Technology, Ulsan, South Korea; ^2^Department of Medical Science, College of Medicine, Catholic Kwandong University, Gangneung, South Korea

**Keywords:** primary motor cortex (M1), decoding algorithm, Kalman filter, long short-term memory recurrent neural network, intracortical brain–machine interface, deep canonical correlation analysis

## Abstract

The control of arm movements through intracortical brain–machine interfaces (BMIs) mainly relies on the activities of the primary motor cortex (M1) neurons and mathematical models that decode their activities. Recent research on decoding process attempts to not only improve the performance but also simultaneously understand neural and behavioral relationships. In this study, we propose an efficient decoding algorithm using a deep canonical correlation analysis (DCCA), which maximizes correlations between canonical variables with the non-linear approximation of mappings from neuronal to canonical variables via deep learning. We investigate the effectiveness of using DCCA for finding a relationship between M1 activities and kinematic information when non-human primates performed a reaching task with one arm. Then, we examine whether using neural activity representations from DCCA improves the decoding performance through linear and non-linear decoders: a linear Kalman filter (LKF) and a long short-term memory in recurrent neural networks (LSTM-RNN). We found that neural representations of M1 activities estimated by DCCA resulted in more accurate decoding of velocity than those estimated by linear canonical correlation analysis, principal component analysis, factor analysis, and linear dynamical system. Decoding with DCCA yielded better performance than decoding the original FRs using LSTM-RNN (6.6 and 16.0% improvement on average for each velocity and position, respectively; Wilcoxon rank sum test, *p* < 0.05). Thus, DCCA can identify the kinematics-related canonical variables of M1 activities, thus improving the decoding performance. Our results may help advance the design of decoding models for intracortical BMIs.

## Introduction

The primary motor cortex (M1) is robustly linked to the kinematic parameters of the upper limbs (Humphrey, 1972; Humphrey and Corrie, 1978; Georgopoulos et al., 1982, 1986; Sergio et al., 2005; Schwartz, 2007; Aggarwal et al., 2008; Vargas-Irwin et al., 2010). This concept provides a basis for decoding information in an intracortical brain–machine interface (BMI), which often harnesses M1 activities to infer continuous movement parameters in order to enable the neural control of external effectors. Intracortical BMIs have largely relied on functional relationships between M1 activities and kinematics (Moran and Schwartz, 1999b; Paninski et al., 2003; Wu et al., 2004; Shanechi et al., 2016; Vaskov et al., 2018). For instance, a number of BMIs have been developed based on a finding that a population vector constructed from the firing activities of a neuronal ensemble can predict the kinematic variables of arm movements, such as direction, speed, position, and velocity (Georgopoulos et al., 1986, 1988; Flament and Hore, 1988; Schwartz et al., 1988; Moran and Schwartz, 1999b; Paninski et al., 2003; Wang et al., 2007). In addition to the population vector, neural representations capturing the shared variability in the population’s neural activity have been demonstrated to be effective in predicting behavioral covariates (Yu et al., 2009; Shenoy et al., 2013; Cunningham and Yu, 2014; Kao et al., 2015). These neural representations can be acquired through unsupervised learning techniques such as principal components analysis (PCA) (Ames et al., 2014; Kaufman et al., 2014), factor analysis (FA) (Yu et al., 2009), and a linear dynamical system (LDS) based latent-state estimation (Kao et al., 2015) and are known to allow a decoder to guarantee stable outputs (Yu et al., 2009; Kao et al., 2013). Such neural representations of neural population activity could help enhance decoding kinematic variables. Decoding models for intracortical BMIs are broadly categorized into two categories. The first category is a generative method that operates based on the generation of neuronal firing activities from kinematic states described by encoding models. The second category is a direct method that operates based on a direct input–output function approximation from neuronal firing activities to kinematic variables (Chapin et al., 1999; Sussillo et al., 2012; Dethier et al., 2013; Ahmadi et al., 2019).

Generative decoding methods designed for BMIs include a population vector algorithm (Georgopoulos et al., 1986; Schwartz and Moran, 2000; Van Hemmen and Schwartz, 2008), a Kalman filter (KF) (Wu et al., 2004, 2006; Gilja et al., 2012; Golub et al., 2014), a point process-based adaptive filter (Wang et al., 2009; Shanechi et al., 2014), and a particle filter (Gao et al., 2001), to name a few. These methods infer kinematic information from observed neuronal activities via encoding models. The performance of generative decoding methods thus substantially depends on the assumptions and appropriateness of encoding models. Furthermore, direct decoding methods designed for BMIs include the Wiener filter (Chapin et al., 1999; Serruya et al., 2002; Fagg et al., 2009; Chhatbar and Francis, 2013; Willett et al., 2013), support vector regression (Kim et al., 2006; Xu et al., 2011), and artificial neural networks (ANNs) (Wessberg et al., 2000; Sanchez et al., 2003). Particularly, ANNs can serve as a direct approximator of a non-linear functional relationship between M1 activities and kinematic variables. Various types of ANNs have been suggested to decode M1 activities, including time-delay neural networks (Kim et al., 2003; Wang et al., 2005), recurrent neural network (RNN) (Sussillo et al., 2012), and echo-state network (Rao et al., 2005). Furthermore, owing to recent breakthroughs in deep learning, using deep neural networks (DNNs) for decoding M1 activities has become plausible (Sussillo et al., 2012; Ahmadi et al., 2019). For instance, a long short-term memory RNN (LSTM-RNN), one of the non-linear models harnessing temporal information in past neural activities, outperformed other decoding models for BMIs (Ahmadi et al., 2019). Despite their high performance, the intricate architectures of DNNs often require a much larger training data to achieve a successful decoding process. Furthermore, recent efforts to record a larger number of neuronal activities (e.g., >1,000 units) demand effective representational spaces of neuronal ensemble activities, which will also reduce the burden of training DNNs (Marblestone et al., 2013).

Considering the advantage of DNNs as a universal non-linear approximator, in the present study, we propose a novel approach for decoding M1 activities to estimate limb kinematics by exploring a joint representational space between M1 activities and kinematics. In this joint space, the representation variables of a neuronal ensemble and kinematic parameters are created in a way to maximize coupling between neuronal and kinematic representation variables. Among the many ways of doing so, we leveraged methods, such as a canonical correlation analysis (CCA), to maximize correlations between these variables. As one of the multivariate statistical methods, CCA maximizes correlations between joint (canonical) variables. A conventional linear canonical correlation analysis (LCCA) builds a linear mapping between a neuronal ensemble and canonical variables (Hotelling, 1936; Anderson, 1984; Friman et al., 2007). However, more informative neuronal canonical variables can be extracted from neuronal ensemble activities by using a non-linear method. A recently developed deep canonical correlation analysis (DCCA) allows us to examine this possibility by approximating a non-linear mapping from neuronal ensemble activities to canonical variables with a DNN (Andrew et al., 2013). Previous non-invasive brain–computer interface (BCI) studies showed the effectiveness of DCCA as a means of feature extraction from electroencephalogram associated with various covariates of interest, such as eye movements and visual stimulus frequencies (Vu et al., 2016; Qiu et al., 2018; Liu et al., 2019). For example, Vu et al. successfully improved the performance of the steady-state visual evoked potential-based BCI using DCCA-based feature extraction (Vu et al., 2016). Although DCCA suffers from the same difficulty in interpreting neural activities as DNNs, canonical variables estimated by DCCA may effectively represent kinematics-related neuronal ensemble activities. Consequently, decoding these canonical variables may achieve a similar or superior performance to decoding original firing rates (FRs) while keeping a decoding model concise.

Based on this hypothesis, the present study aims to investigate how hand velocity information is represented in canonical variables found by LCCA or DCCA and to compare those representations with other neural representations (PCA, FA, and LDS) extracted from naïve ensemble FRs (Z_E–FR_). Moreover, we aim to investigate the performance of decoding hand velocity information from the five types of neuronal representations (E-FR, PCA, FA, LCCA, and DCCA) using one of the two types of decoders, i.e., LKF and LSTM-RNN. Additionally, we examine whether DCCA yields better velocity decoding performance compared to a neural dynamical filter (NDF), which is a linear mapping model to predict kinematic variables from LDS-based latent states (Kao et al., 2015). In this study, we apply various decoding methods to the data of M1 firing activity and hand movements in two non-human primates that performed a 2D reaching task with one arm.

## Materials and Methods

### Datasets

**FIGURE 1:**
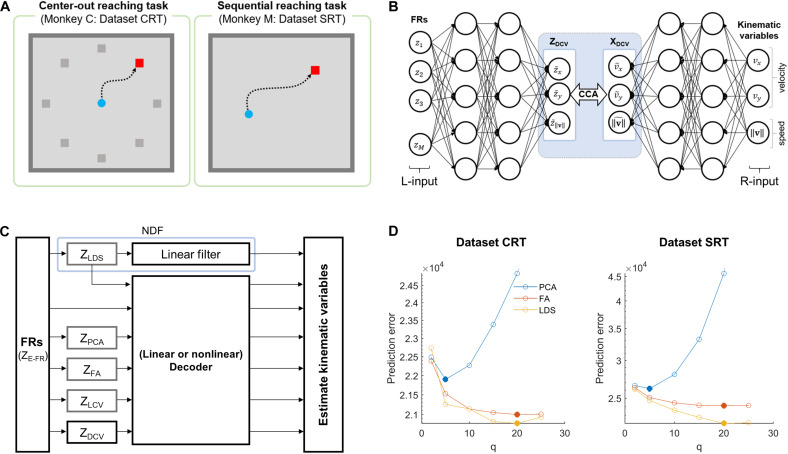
Simulation overview for assessing the effects of DCCA on two decoders. **(A)** Behavioral tasks for each dataset. The left panel denotes a center-out reaching task which the monkey C performed, and the right panel is a sequential reaching task which the monkey M performed. **(B)** The schematic diagram depicts the DCCA between firing rates and kinematic variables. The left inputs (L-input) of the networks indicate the naïve firing rates and the right inputs (R-input) of the networks denote the kinematic variables: *x*- and *y*-velocity, and speed. A dotted-line box between the networks denotes a canonical correlation analysis (CCA) between the left-canonical variables (Z_DCV_) and the right-canonical variables (X_DCV_). **(C)** The block diagram depicts a simulation paradigm for a comparative study of decoding. **(D)** Prediction errors for the state dimensionalities (*q*) of each dataset. The filled circle denotes proper dimensionality corresponding to the minimum prediction error for each dimensionality reduction method (Yu et al., 2009). Each color code denotes the dimensionality reduction method.

The two datasets used in this study are available on a neuroscience data depository, called the Collaborative Research in Computational Neuroscience (Flint et al., 2012; Lawlor et al., 2018; Perich et al., 2018). Each dataset includes the cortical firing activity and hand movement recordings, which were acquired from a non-human primate performing an arm reaching task on 2D spaces with one arm (see Figure 1A). The dataset CRT (center-out reaching task) of Flint et al. includes M1 activities for monkeys to perform a center-out reaching task to acquire eight different targets that were placed at 45° intervals around a circle with their home placed on the center (Flint et al., 2012). The dataset SRT (sequential reaching task) of Lawlor et al. (2018) includes M1 and dorsal premotor cortical activities during a sequential reaching task, where a series of targets were randomly displayed on 2D spaces (Lawlor et al., 2018; Perich et al., 2018). All cortical activities were extracellularly recorded by a 128-channel acquisition system (Cerebus, Blackrock Microsystems, Inc., Salt Lake City, UT, United States) through 96-channel silicon microelectrode arrays chronically implanted in the arm area of M1.

In this study, we analyzed only M1 activities to develop and test decoders. The spike trains of each neuron were binned with a non-overlapping window of 50 ms to maximize the mutual information between neural FRs and kinematics (Paninski et al., 2003; Suminski et al., 2010). FRs were estimated by spike counts within the bin divided by its size (i.e., 50 ms). We also square-root-transformed the FRs in each bin to make them more normally distributed for linear decoding models (Schwartz and Moran, 1999). Then, we performed a Gaussian kernel smoothing process to reduce temporal noise of individual unit activities, where the kernel standard deviation (SD) was determined according to Yu et al. (2009) (*SD* = 80 ms in the dataset CRT, *SD* = 140 ms in the dataset SRT). An instantaneous hand position was converted into the velocity and its absolute value (speed). This kinematic combination (velocity and speed) is shown to be appropriate predictors for establishing tuning models (Rasmussen et al., 2017). Using the velocity and speed, we calculated the goodness of fit (*r*^2^) of a linear tuning model for each neuron, which was designed based on the cosine tuning model (Moran and Schwartz, 1999a), expressed by: z(*t*) = β_0_ + β_1_v(*t*) + β_2_v(*t*) + *ϵ*(*t*) where z(*t*) is FRs, β_0_,β_1_, and β_2_ are model coefficients, and v(*t*) and v(*t*) denote a vector of velocity and its norm (speed) at time *t*, respectively. Then, we selected the neurons with *r*^2^ > 0.01, where the threshold of *r*^2^ (>0.01) was empirically determined. A total of 155 and 63 neurons passed these criteria in the datasets CRT and SRT, respectively. The datasets CRT and SRT included 175 and 496 successful trials, respectively, in which animals successfully acquired the targets during the tasks. To build and validate decoders, the first 75% of the trials were used for training and the remaining 25% of the trials were used for testing: the training and testing sets of the dataset CRT contained 131 and 44 trials, respectively, and those of the dataset SRT contained 372 and 124 trials, respectively. Every parameter estimation of the models built in this study (see below) was performed using the training set only.

### Neural Representation Extraction via Supervised Learning Methods

#### Linear Canonical Correlation Analysis

Canonical correlation analysis is one of the multivariate statistical methods that extracts joint canonical variables from random vectors *z* and *x*. In this study, *z* and *x* correspond to the FRs [*z*_1_,*z*_2_,…,*z*_*M*_]^T^ ∈ *ℝ*^*m*×1^ from *m* neurons and the hand kinematics [*x*_1_,*x*_2_,*x*_3_]^T^ ∈ *ℝ*^3×1^, where *x*_1_ and *x*_2_ denote the velocity of the *x*- and *y*-coordinates, respectively, and *x*_3_ denotes the speed. An LCCA seeks linear mappings from *z* and *x* to canonical variables by maximizing correlations between canonical variables (Hotelling, 1936; Anderson, 1984; Friman et al., 2007). The canonical coefficients {α,β} on these linear mappings are defined as

(1) $$\left\{\alpha^{*},\beta^{*}\right\}=\underset{\alpha^{*},\beta^{*}}{\text{argmax}}\rho \left(\alpha^{\text{T}}\text{z},\beta^{\text{T}}\text{x}\right)$$

(2) $$=\underset{\alpha^{*},\beta^{*}}{\text{argmax}}\frac{\alpha^{\text{T}}{\text{Σ}}_{\text{ZX}}\beta }{\sqrt{\alpha^{\text{T}}{\text{Σ}}_{\text{Z}}\alpha \cdot \beta^{\text{T}}{\text{Σ}}_{\text{X}}\beta }}$$

where ρ(⋅) denotes a function of the correlation between canonical variables. Σ_*Z*_ and Σ_*X*_ are the covariance matrices of centralized data $\bar{\text{z}}$ and $\bar{\text{x}}$, respectively, and Σ_*ZX*_ is the sample cross-covariance matrix. To make the canonical coefficients scale-free, the denominator is constrained to have unit variance, such that

(3) $$\left\{\alpha^{*},\beta^{*}\right\}=\underset{\alpha^{\text{T}}{\text{Σ}}_{\text{Z}}\alpha =\beta^{\text{T}}{\text{Σ}}_{\text{X}}\beta =1}{\text{argmax}}\alpha^{\text{T}}{\text{Σ}}_{\text{ZX}}\beta$$

The singular value decomposition is used to derive α^∗^ and β^∗^. Using these variables, the canonical variables of *z* and *x* can be estimated by

(4) $${\hat{u}}_{Z}=\alpha^{*T}\bar{\text{z}}$$

(5) $${\hat{u}}_{X}=\beta^{*T}\bar{\text{x}}$$

By using the eigenvectors corresponding to the largest eigenvalues, we repeatedly computed a pair of canonical variables, {${\hat{u}}_{Z},{\hat{u}}_{X}$}, until the number of pairs equals *m* or 3. For convenience, we call the linear neural (${\hat{u}}_{Z}$) and kinematic (${\hat{u}}_{X}$) canonical variables Z_LCV_ and X_LCV_, respectively.

#### Deep Canonical Correlation Analysis

A DCCA is one of the advanced CCA methods based on DNNs. DCCA finds non-linear mappings from *z* and *x* to canonical variables through stacked non-linear transformation layers, as shown in Figure 1B (Andrew et al., 2013). The non-linear mappings $f_{\text{z}}^{l}\left(z\right)$ and $f_{\text{x}}^{l}\left(x\right)$ are defined as

(6) $$f_{\text{z}}^{l}\left(z\right)=\sigma \left({\text{W}}_{l}^{\left(Z\right)}{\text{u}}_{l-1}^{\left(Z\right)}+{\text{b}}_{l}^{\left(Z\right)}\right)\in {\mathbb{R}}^{m\times 1}$$

(7) $$f_{\text{x}}^{l}\left(x\right)=\sigma \left(W_{l}^{\left(X\right)}{\text{u}}_{l-1}^{\left(X\right)}+{\text{b}}_{l}^{\left(X\right)}\right)\in {\mathbb{R}}^{3\times 1}$$

where $W_{l}^{\left(\cdot \right)}$ denotes a matrix of weights at the *l-th* layer, ${\text{u}}_{l-1}^{\left(\cdot \right)}$ is the output vector from the (*l−*1)*-th* layer, ${\text{b}}_{\text{l}}^{\left(\cdot \right)}$ is a vector of biases at the *l-th* layer, and σ(⋅) is a non-linear function. A parameter set θ, which includes *W* and **b**, is estimated by maximizing correlations between functional outputs as follows:

(8) $$\text{arg}\max_{\theta_{\text{Z}}^{*},\theta_{\text{X}}^{*}}\rho \left(f_{Z}\left(z,\theta_{Z}\right),f_{X}\left(x,\theta_{\text{X}}\right)\right)$$

To seek $\theta_{\text{Z}}^{*}$ and $\theta_{\text{X}}^{*}$, the backpropagation algorithm is used to optimize parameters *W* and **b** based on the gradient of ρ(⋅). The parameters in each layer are initialized in advance through a pretraining process using a denoising autoencoder (Vincent et al., 2008). The deep neural canonical variables can be computed as ${\hat{o}}_{Z}=f_{Z}\left(z,\theta_{Z}\right)$, and the deep kinematic canonical variables can be computed as ${\hat{o}}_{X}=f_{X}\left(x,\theta_{\text{X}}\right)$. In that case, we call the deep neural (${\hat{o}}_{Z}$) and kinematic (${\hat{o}}_{X}$) canonical variables Z_DCV_ and X_DCV_, respectively.

In addition to θ, we also need to optimize the hyperparameters of DNNs, for which we employed the Bayesian optimization method (Ahmadi et al., 2019). To optimize the hyperparameters, we empirically preset the range for each parameter: the number of nodes in a layer ∈ {2^4^, 2^5^, …, 2^10^}, the number of layers ∈ {1, 2, …, 4}, an encoder and decoder batch size ∈ {2^5^, 2^6^, …, 2^8^}, a learning rate ∈ {1e−5, 1e−4, …, 1e−2}, a regularization parameter for each view ∈ {1e−6, …, 1e−1}, a weight decay parameter (or an L_2_ regularization parameter) ∈ {1e−6, …, 1e−1}, and a trade-off parameter ∈ {1e−6, …, 1e−1}. While other parameters determine the learning and architecture of a general DNN, the trade-off parameter is used for regularizing correlations with a quadratic penalty, uniquely associated with DCCA. The Bayesian optimization is iteratively performed 1,000 times to select reliable parameters. Table 1 shows the optimized hyperparameters obtained in this study for each dataset using the publicly available MATLAB toolbox for the DCCA (Wang et al., 2015).

**TABLE 1:** Optimized hyperparameters for DCCA with respect to each dataset.

	Hyperparameters	CRT	SRT
**Function of *Z***	# nodes (*z*)	1024	256
	# layers (*z*)	2	2
	RCOV (*z*)	0.04	0.08
**Function of *X***	# nodes (*x*)	1024	64
	# layers (*x*)	3	3
	RCOV (*x*)	0.04	0.04
**Common**	Batch size (encoder, autoencoder)	256/256	128/128
	Batch size (decoder, autoencoder)	64/64	512/512
	*L*_2_ regularization	6.8e−04	3.7e−04
	η	0.01	0.01
	λ	0.01	0.02

*Hidden layer activator is fixed as a sigmoid function.η, learning rate; λ, trade-off parameter; RCOV, regularization parameter for the total layers.*

### Neural Representation Extraction via Unsupervised Learning Methods

For the purpose of comparison, we also extracted low-dimensional representations of neural population firing activity using several methods, including PCA, FA, and LDS, which are widely used in intracortical BMIs. Below we describe each method briefly.

#### Principal Component Analysis

We applied principal component analysis (PCA) to the FR data of all neuronal units. A Gaussian kernel smoothing process was used as preprocessing for FRs before applying PCA to avoid a case where neurons with highly fluctuating firing rates influenced decoding (Yu et al., 2009). Then, we extracted principal components (PCs) of FR using PCA from the training data. Note that PCA was performed on a single trial basis rather than trial-averaged data in order to focus on covariance between neuronal units in single trials. To determine the number of PCs that would be included in the set of neural representations, we followed the procedure proposed by Yu et al. (2009). Briefly, using the eigenvectors obtained from the training set, we extracted all PCs (i.e., as many as neuronal units) for the testing set, which were then sorted according to the magnitude of corresponding eigenvalues in a descending way. Afterward, we selected the first 5 PCs and reconstructed FRs from them. The mean absolute error between true FRs and reconstructed FRs was calculated. We kept adding the next 5 PCs, reconstructing FRs and calculating error in the same way as above. As a result, the minimum reconstruction error was achieved with the first 5 PCs for both datasets of CRT and SRT (Figure 1D), which constituted neural representations by PCA, denoted as Z_PCA_. Note that the smoothing process was applied again to the final set of PCs before decoding.

#### Factor Analysis

A factor analysis (FA) allows us to find low-dimensional latent factors to elucidate shared variability among the population activities (Santhanam et al., 2009). Again, we performed the smoothing to FRs before applying FA. To estimate latent factors from FRs, we adopted the FA method proposed by Yu et al., which adjusted FA for neural data (Yu et al., 2009; Kao et al., 2015). Then, in a similar way to PCA, we determined the number of factors included in a set of neural representations using the reconstruction error of the testing set. We found the minimum error with 20 factors for both CRT and SRT datasets, which were further used as the set of neural representations by FA, denoted as Z_FA_.

#### Linear Dynamical System for M1 States

Observed neuronal population activity can be interpreted as a noisy observation of low-dimensional and dynamical neural states (Shenoy et al., 2013; Kao et al., 2015). Using the LDS-based neural state estimation approach proposed by Kao et al. (2015), we estimated dynamic neural latent states from the population activity. We determined the dimensionality of neural states using the procedure above based on reconstruction error. We set the dimensionality to 20 for both CRT and SRT datasets, with which the minimum reconstruction error was achieved. A vector of these neural state was used as neural representations by LDS, denoted as Z_LDS_. Note that we used a linear filter [formally called a neural dynamical filter (NDF)] instead of Kalman filter when decoding Z_LDS_ because Z_LDS_ already represented latent dynamics of neural activity such that state estimation of Kalman filter might not be suitable for it.

### Neural Representation Analysis

We first examined Pearson correlations between canonical variables; $\rho \left({\hat{u}}_{Z},{\hat{u}}_{X}\right)$ or $\rho \left({\hat{o}}_{Z},{\hat{o}}_{Z}\right)$. Both canonical variables of neural FRs (${\hat{u}}_{Z}$ or ${\hat{o}}_{Z}$) are supposed to adequately represent kinematic information because they are highly correlated with the canonical variables of kinematic parameters (${\hat{u}}_{X}$ or ${\hat{o}}_{X}$), provided that the linear or non-linear mappings of LCCA or DCCA are appropriately built. To validate this assumption, we performed a tuning analysis of not only the neural canonical variables but also other neural representations using a linear regression model, in which the tuning quality of each neural representation with respect to velocity and speed was analyzed. The temporal linear regression model of a single neural representation (*z*) to the kinematic parameters (*x*) was given as **z**(*t*) = β_0_ + β**x**(*t*) + ϵ(*t*) where β_0_ and β denote coefficients and ϵ(*t*) is the error term at time *t* (Schwartz and Moran, 1999, 2000; Paninski et al., 2003; Rasmussen et al., 2017). The tuning quality of a neural representation was assessed by the goodness-of-fit (*r*^2^) of the tuning model. In addition to this, we also computed the decoding performance using each neural representation in the training data with a linear Kalman filter. The decoding performance was measured by the mean absolute error between actual and decoded velocity from the training data. Finally, we assessed the predictive performance of each of the five neural representations above during training using both the goodness-of-fit of the tuning model and the training error.

### Decoding Algorithms

#### Kalman Filter

A linear Kalman filter (LKF) is one of the popular generative decoding methods based on the linear dynamical system (Wu et al., 2006). LKF follows a first-order Markov rule, such that a state vector (velocity and speed) ^*x*^*_*t*_* at time *t* evolves from x*_*t*_*_–__1_ at time *t*−1. In this study, the state vector corresponds to the kinematic parameter vector. The system model, which describes the state transition, and the observation model, which describes the generation of observation *o*_*t*_ from *x*_*t*_, are given by

(9) $${\text{x}}_{\text{t}}=A{\text{x}}_{t-1}+Q_{t-1}$$

(10) $${\text{o}}_{\text{t}}=H{\text{x}}_{\text{t}}+V_{\text{t}}$$

where *A* denotes the system model parameter matrix, *H* is the observation model parameter matrix, and *Q* and *V* are the process and observation noise following a Gaussian distribution, respectively. The neural observation vector **o**_*t*_ can be either the Z_E–FR_ vector (**z**_*t*_) or the vector of the other neural representations. To predict **x**_*t*_, we initialized **x**_0_ = **0** at the beginning of every trial after converging the Kalman gain to its steady state in advance (Dethier et al., 2013).

#### Long Short-Term Memory in Recurrent Neural Networks

An LSTM-RNN based on an RNN architecture has been well suited in predicting kinematics from neuronal activities (Ahmadi et al., 2019). The components of LSTM-RNN are enumerated as follows: *c* is a memory cell, **f** is a forget gate, and **i** and **o** are input and output gates, which correspond to *ℝ^l^*, where *l* denotes the number of hidden units. LSTM-RNN operates by regulating the information flow with these gates via the cell. Given *W* as a matrix of weights with respect to the recurrent connection or input/output, **h** as a vector of the hidden layer, and **b** as a vector of biases, each gate can be calculated by

(11) $${\text{f}}_{\text{t}}=\sigma_{sigmoid}\left(W_{f,z}{\text{y}}_{\text{t}}+W_{f,l}{\text{h}}_{t-1}+{\text{b}}_{\text{f}}\right)$$

(12) $${\text{i}}_{\text{t}}=\sigma_{sigmoid}\left(W_{i,z}{\text{y}}_{\text{t}}+W_{i,l}{\text{h}}_{t-1}+{\text{b}}_{\text{i}}\right)$$

(13) $${\text{o}}_{\text{t}}=\sigma_{sigmoid}\left(W_{o,z}{\text{y}}_{\text{t}}+W_{o,l}{\text{h}}_{t-1}+{\text{b}}_{\text{o}}\right)$$

where the input vector **y** is either the Z_E–FR_ vector (**z**_*t*_) or the vector of the other neural representations at time *t* and σ_*sigmoid*_(⋅) denotes the sigmoidal activation function. The subscripts indicate the corresponding gates and their recurrent connection. The information flow of the cell memory can be updated by

(14) $${\text{c}}_{\text{u}}=\sigma_{tanh}\left(W_{c,z}{\text{z}}_{\text{t}}+W_{c,l}{\text{h}}_{t-1}+{\text{b}}_{\text{c}}\right)$$

(15) $${\text{c}}_{\text{t}}=f_{\text{t}}⊗{\text{c}}_{t-1}+{\text{i}}_{\text{t}}⊗{\text{c}}_{\text{u}}$$

(16) $${\text{h}}_{\text{t}}={\text{o}}_{\text{t}}⊗\sigma_{tanh}\left({\text{c}}_{\text{t}}\right)$$

where σ_*tanh*_(⋅) denotes the hyperbolic tangent function and ⊗ denotes the element-wise product. To train LSTM-RNN, we utilized the Adam optimizer built-in MATLAB deep learning toolbox. The hyperparameters of LSTM-RNN were optimized by the Bayesian optimizer in the same way as DCCA. The Bayesian optimizer performed an objective function evaluation 500 times. In our analysis, we set the gradient decay factor as 0.95 and the squared gradient decay factor as 0.99. Then, the training batches were shuffled at every epoch for the training efficiency. Table 2 shows the optimized hyperparameters for LSTM-RNN.

**TABLE 2:** Optimized hyperparameters with respect to the representation pairs for LSTM-RNN (CRT/SRT dataset).

	Z_E–FR_	Z_PCA_	Z_FA_	Z_LDS_	Z_LCV_	Z_DCV_
No. nodes	35/40	15/64	15/64	15/15	15/64	35/64
Mini-batch size	128/16	16/256	16/32	128/64	128/32	128/32
RCOV	0.01/0.01	0.05/1e−03	0.02/0.09	0.08/0.09	0.1/0.1	0.1/0.1
η	6e−03/1e−04	0.01/1e−04	0.01/1e−04	1e−04/1e−04	0.01/1e−04	1e−03/1e−04

*η, learning rate; RCOV, regularization parameter; no. hidden layers {1}, gradient decay factor {0.95}, squared gradient decay factor {0.95}, and activation function {logistic sigmoid} are fixed.*

### Decoding Performance Evaluation

To evaluate the effects of CCA on decoding, we composed three representations of neuronal activities: Z_E–FR_, Z_PCA_, Z_FA_, Z_LDS_, Z_LCV_, and Z_DCV_ (see Figure 1C). In this study, we performed decoding to predict the hand velocity X_VEL_ from each neural representation using LKF and LSTM-RNN.

For the evaluation of the decoding performance, we measured the decoding error by the Euclidean distance between the actual and predicted kinematic parameters. The decoding error was measured for the hand velocity *v* and hand position *p*, which were reconstructed from the cumulated velocity for each trial. The decoding error of the *i*-th trial was calculated as

(17) $${\text{e}}_{\text{i}}=\frac{1}{n}\sum_{t=1}^{n}e\left(t\right)$$

where *e*(*t*) is an absolute instantaneous error, $e\left(t\right)=\left|\text{v}\left(t\right)-\hat{\text{v}}\left(t\right)\right|$ or $e\left(t\right)=\left|\text{p}\left(t\right)-\hat{\text{p}}\left(t\right)\right|$ at time *t*, and *n* is the number of samples in the *i*-th trial. To compare the decoding performance between the neural representations (Z_E–FR_, Z_PCA_, Z_FA_, Z_LDS_, Z_LCV_, and Z_DCV_), we applied the Friedman test to evaluate the effects of decoder inputs in accordance with the types of decoders (LKF and LSTM-RNN). For the Friedman test, the dependent variables consist of a decoding error, and the factors include the decoder input and decoder type. We also performed a *post hoc* statistical analysis using the Bonferroni correction (*p* < 0.05).

## Results

**FIGURE 2:**
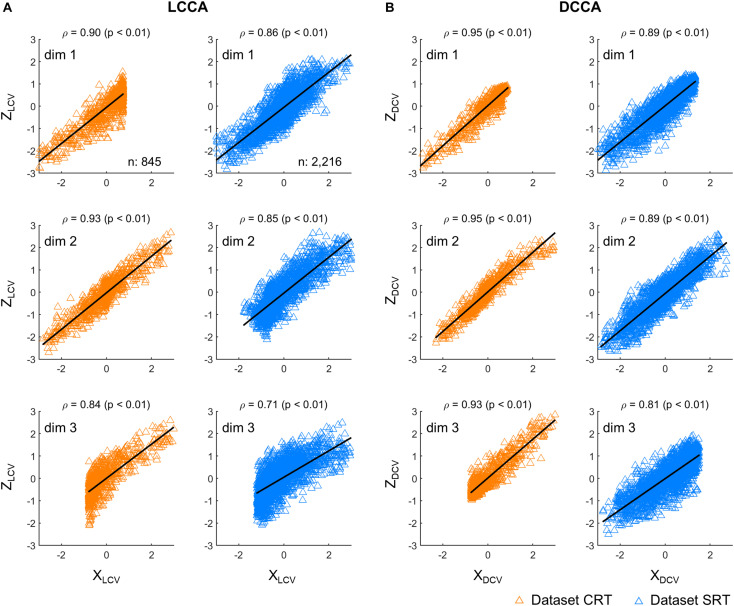
Correlations between canonical variables. **(A)** Correlations between canonical variables extracted by LCCA (Z_LCV_ and X_LCV_). **(B)** Correlations between canonical variables extracted by DCCA (Z_DCV_ and X_DCV_). The upward-pointing triangles denote the samples per time step of the canonical variables. ρ denotes the Pearson’s correlation coefficient and *p* indicates to exist a significant linear regression relationship between *X* and *Z*. Each row corresponds to each dimensionality of the canonical variables. The orange triangles denote the dataset CRT and the blue triangles represent the dataset SRT.

First, we investigated correlations between the neural and kinematic canonical variables. Figure 2 depicts correlations between the canonical variables, each extracted from firing rates (*Z*) and kinematics (*X*), respectively. The canonical variables were obtained from the testing set either by using LCCA or DCCA. Correlations were calculated between the corresponding pairs of neural and kinematic canonical variables, where a total of three pairs were determined by the number of kinematic parameters. DCCA resulted in higher correlations than LCCA for every dataset: the correlation coefficients for the dataset CRT ranged from 0.93 to 0.95 using DCCA and from 0.84 to 0.90 using LCCA (*p* < 0.01, Wilcoxon rank sum test), and those for the dataset SRT ranged from 0.81 to 0.89 using DCCA and from 0.71 to 0.86 using LCCA (*p* < 0.01, Wilcoxon rank sum test).

Next, we examined the tuning of neuronal FRs and neural representations (Z_E–FR_, Z_PCA_, Z_FA_, Z_LDS_, Z_LCV_, and Z_DCV_) concerning kinematic parameters (X_VEL_) using the testing set. The quality of tuning was measured by the *r*^2^ of the linear regression model with X_VEL_ as the regressors (see Section “Neural Representation Analysis”).

**FIGURE 3:**
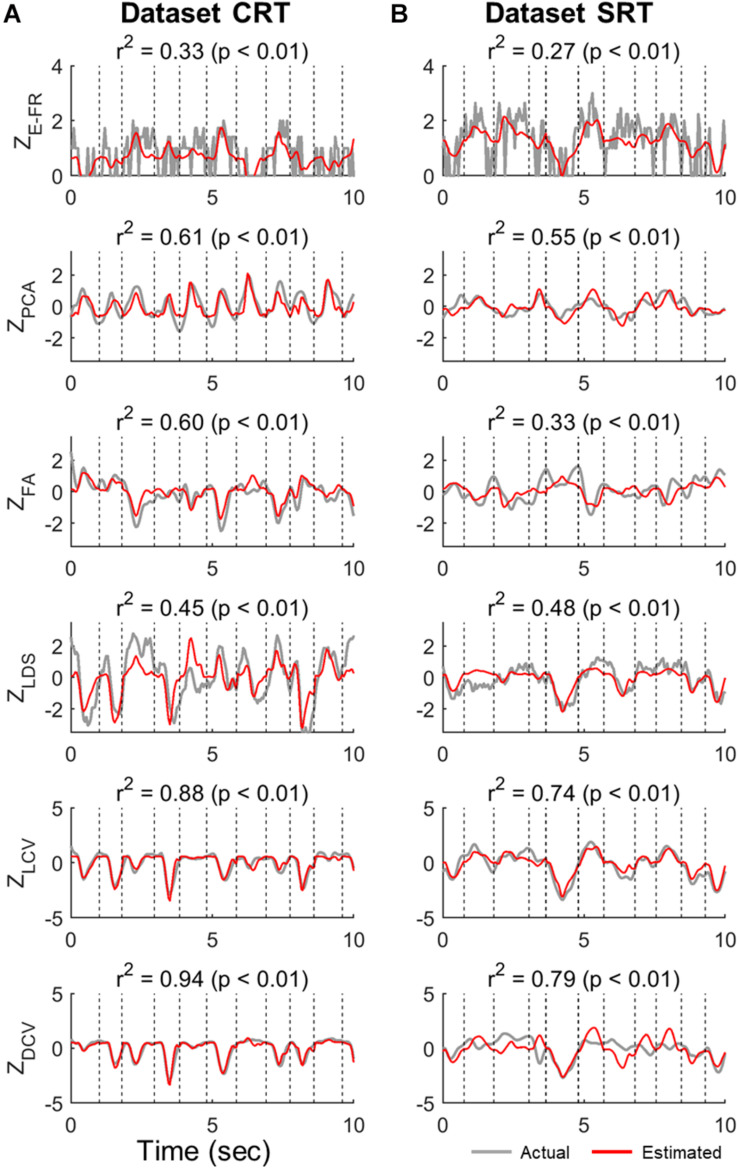
Estimation of neural representations by linear velocity tuning models (testing data). Single traces of the actual neural representations over time in each trial of the test data (gray lines) are superimposed by the corresponding estimates by the linear velocity tuning model (red lines). Here, we present the representative traces of neural representations that were most accurately estimated by the linear velocity tuning models yielding the highest *r*^2^, where *r*^2^ denotes the goodness-of-fit of the linear velocity tuning model. The top row indicates the estimation of Z_E–FR_ in each dataset (CRT and SRT). From the second to fourth rows are the estimations of Z_PCA_, Z_FA_, and Z_LDS_ in each dataset. The bottom two rows denote the estimation of Z_LCV_ and Z_DCV_. Column **(A)** and **(B)** correspond to dataset CRT and SRT, respectively.

Figure 3 shows the examples of the actual values of Z_E–FR_, Z_LCV_, and Z_DCV_ and the estimated values by the linear velocity tuning model. For Z_E–FR_, we selected the neuron whose FRs was most accurately estimated by the model (i.e., the highest value of *r*^2^). Among the neural representations analyzed here, the linear model tracked the variation of Z_DCV_ most accurately yielding the highest *r*^2^ (Friedman test, Bonferroni correction, *p* < 0.05). Notably, the linear model can estimate even time-invariant parts of Z_DCV_ (see the bottom row of Figure 3), which often spanned over multiple trials, even though X_VEL_ varied during these periods.

**FIGURE 4:**
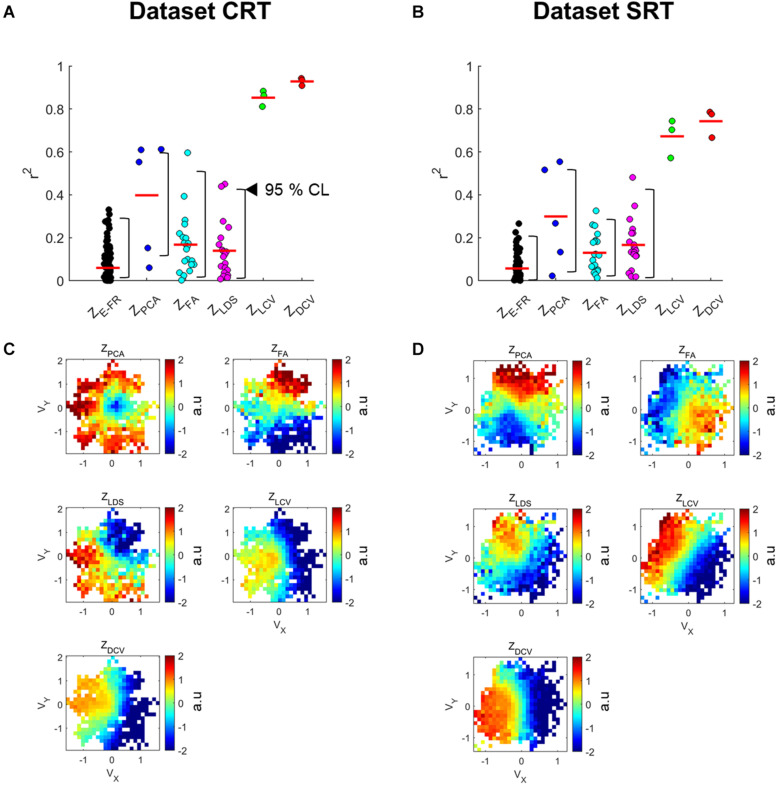
Velocity tuning properties of neuronal canonical variables estimated by the neural representations. **(A,B)** The points denote the linear velocity tuning quality (*r*^2^) for all dimensions of the input variables (Z_E–FR_, Z_PCA_, Z_FA_, Z_LDS_, Z_LCV_, and Z_DCV_). The red horizontal line denotes the averaged *r*^2^ of all dimensions. Black left-pointing pointer denotes a 95% confidence level of each neural representation’s *r*^2^. **(C,D)** Each panel depicts the topographical map of the input variable to the kinematic variables, such as velocity (*v*). In this case, each panel corresponds to the best-tuned dimensionality showing high *r*^2^.

Figures 4A,B depict the distributions of *r*^2^ for Z_E–FR_, Z_LCV_, and Z_DCV_ in the datasets CRT and SRT, respectively. The mean values of *r*^2^ for Z_DCV_ (0.93 in the dataset CRT and 0.74 in the dataset SRT) and Z_LCV_ (0.85 in the dataset CRT and 0.67 in the dataset SRT) were considerably higher than those for Z_PCA_ (0.40 in the dataset CRT and 0.30 in the dataset SRT), Z_FA_ (0.17 in the dataset CRT and 0.13 in the dataset SRT), and Z_LDS_ (0.14 in the dataset CRT and 0.17 in the dataset SRT) (Friedman test, multiple comparison with Bonferroni correction, *p* < 0.05). Moreover, the neural canonical variables found by DCCA (Z_DCV_) was more tuned to velocity than those by LCCA (Z_LCV_) (Wilcoxon rank sum test: *p* = 0.02 in the dataset CRT, *p* = 0.04 in the dataset SRT). Moreover, the neural canonical variables found by DCCA (Z_DCV_) was more tuned to velocity than those by LCCA (Z_LCV_). Figures 4C,D depict the topographical maps of the neural canonical variables showing high *r*^2^ in the 2D velocity space. Although Z_LCV_ and Z_DCV_ were created to maximize correlations with the canonical variables of kinematics, not kinematics *per se*, they showed tuning with the actual velocity.

**FIGURE 5:**
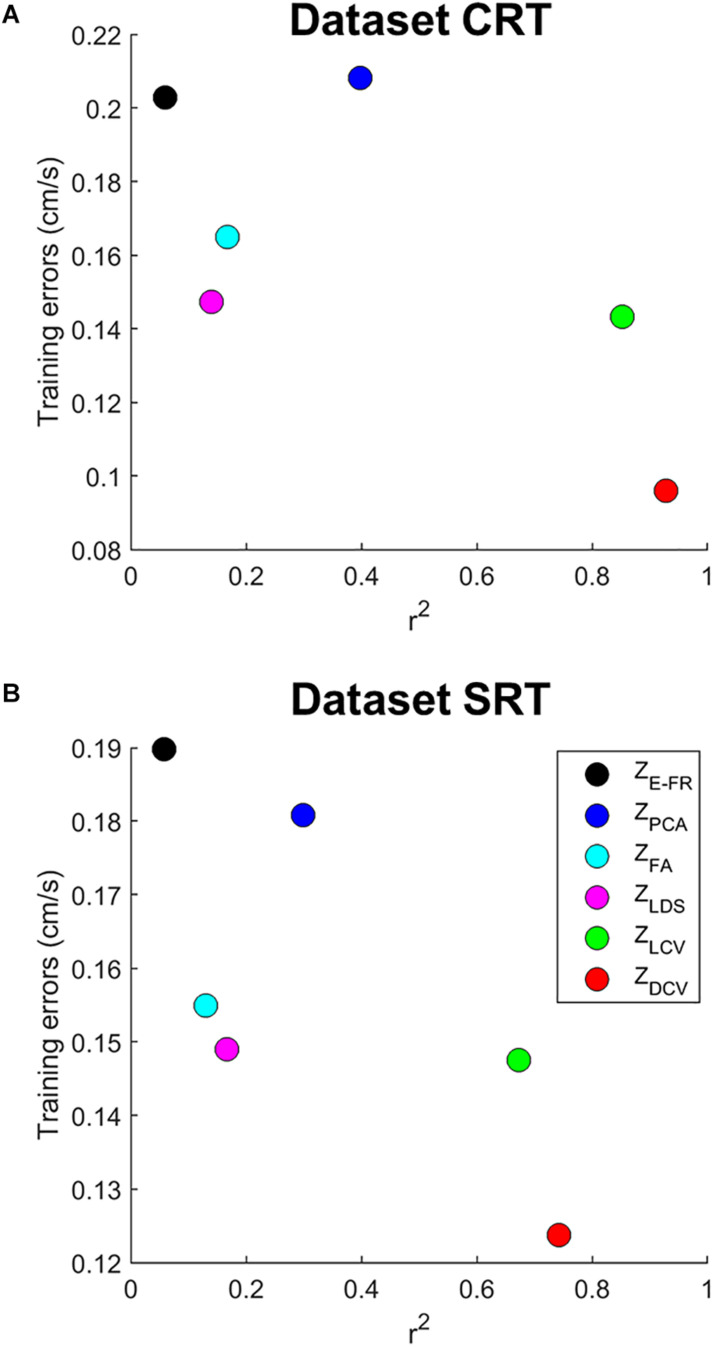
The relationship between training error and average *r*^2^ of velocity-tuning for each dimensionality of the neural representations. Each colored circle corresponds to the mean of *r*^2^ and training error for a neural representation (Z_E–FR_, Z_PCA_, Z_FA_, Z_LDS_, Z_LCV_, and Z_DCV_). The **(A)** top and **(B)** bottom panel correspond to the datasets CRT and SRT.

We then examined both the training error and average *r*^2^ of each neural representation, as shown in Figure 5. It reveals that Z_DCV_ yielded not only the highest *r*^2^ but also the lowest training error (0.09 in the dataset CRT, 0.12 in the dataset SRT). Although we also observed relatively low training error using neural representations of FA and LDS, the average *r*^2^ of them were not high compared to those of CCA.

**FIGURE 6:**
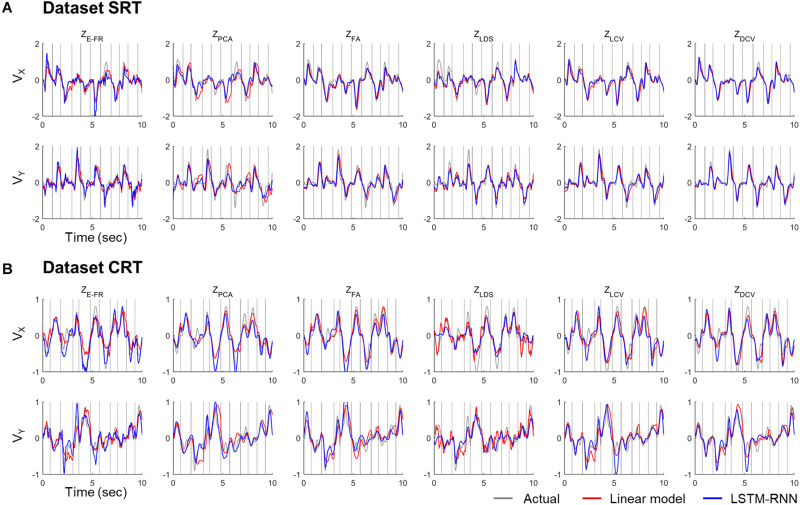
Decoded velocity trajectory from each pair of the variables (testing data). Each column denotes the decoded (*X*- and *Y*-axis) velocity trajectories according to the predictors: Z_E–FR_, Z_PCA_, Z_FA_, Z_LDS_, Z_LCV_, and Z_DCV_. The solid gray lines denote the actual velocity, and the solid red and blue lines depict the outputs of linear model and LSTM-RNN, respectively. For linear model, LKF was used for Z_E–FR_, Z_PCA_, Z_FA_, Z_LCV_, and Z_DCV_, whereas NDF (linear filter) was used for Z_LDS_. The vertical gray lines denote boundary between trial intervals for the reaching. The top **(A)** and bottom **(B)** panel correspond to the datasets CRT and SRT.

**FIGURE 7:**
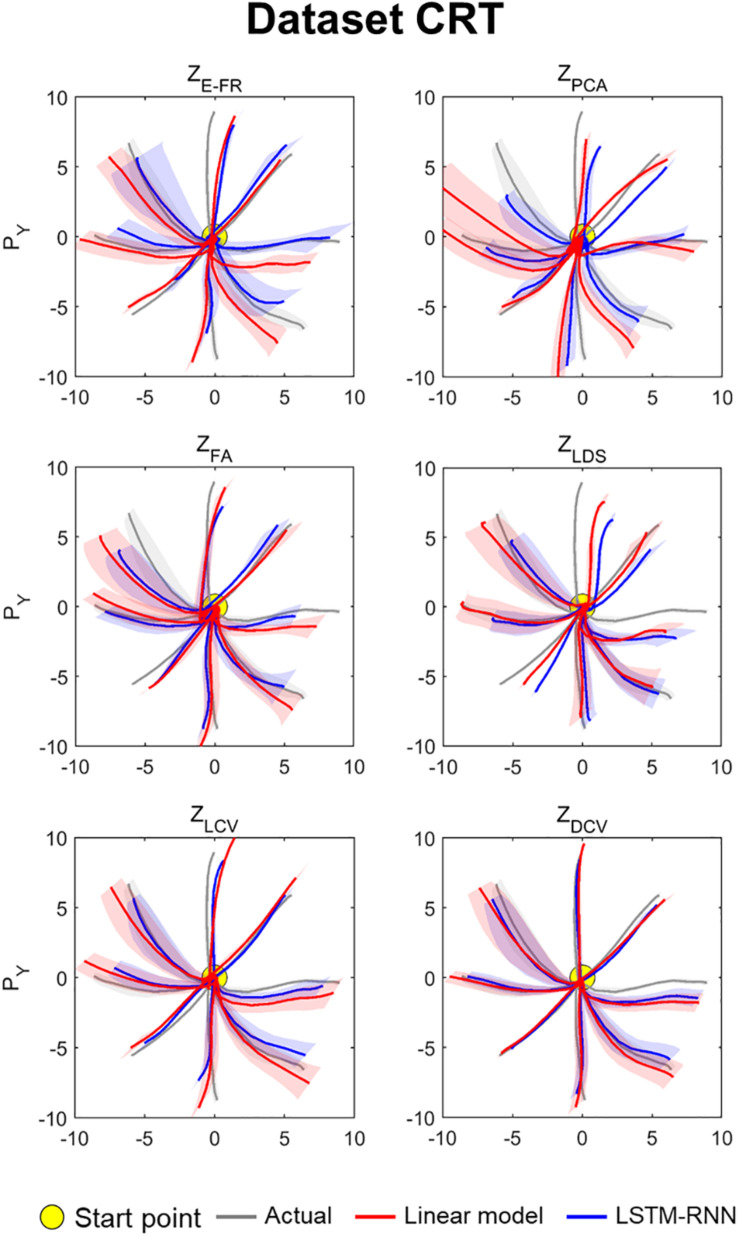
Reconstructed position trajectory in the dataset CRT (testing data). Each panel denotes the reconstructed position trajectories according to the predictors: Z_E–FR_, Z_PCA_, Z_FA_, Z_LDS_, Z_LCV_, and Z_DCV_. Solid gray lines denote the true position trajectories, red lines denote the position trajectories reconstructed from the output of linear model, and blue lines denote the position trajectories from the output of LSTM-RNN. For linear model, LKF was used for Z_E–FR_, Z_PCA_, Z_FA_, Z_LCV_, and Z_DCV_, whereas NDF (linear filter) was used for Z_LDS_. The filled yellow circle denotes the home position (0, 0) from which non-human primates started to move their hands. Solid lines denote the averaged position trajectories across the trials, and shaded lines denote the standard errors across 44 trials with respect to each direction.

The decoding performance was evaluated for each combination of neural representations and decoders (see Figure 1). Figure 6 depicts the true and decoded velocity trajectories for each combination. The results show that decoding Z_DCV_ produced the most accurate prediction of velocity (Friedman test with Bonferroni correction, *p* < 0.05. See Tables 3, 4). Figure 7 depicts the true and reconstructed position trajectories in the dataset CRT. When decoding Z_E–FR_ and Z_LCV_, LSTM-RNN reconstructed the hand position more accurately than LKF. However, when decoding Z_DCV_, there was no apparent difference between the decoders. Z_DCV_ also led to the smallest variance of the reconstructed trajectories [variance, Z_E–FR_: *x*-pos = 0.83, *y*-pos = 0.82; Z_PCA_: *x*-pos = 1.00, *y*-pos = 1.01; Z_FA_: *x*-pos = 0.83, *y*-pos = 0.80; Z_LDS_ (NDF): *x*-pos = 0.71, *y*-pos = 0.74; Z_LCV_: *x*-pos = 0.74, *y*-pos = 0.80; Z_DCV_: *x*-pos = 0.61, *y*-pos = 0.62 when using LKF, whereas Z_E–FR_: *x*-pos = 0.87, *y*-pos = 0.77; Z_PCA_: *x*-pos = 0.75, *y*-pos = 0.79; Z_FA_: *x*-pos = 0.70, *y*-pos = 0.65; Z_LDS_: *x*-pos = 0.78, *y*-pos = 0.77; Z_LCV_: *x*-pos = 0.60, *y*-pos = 0.60; Z_DCV_: *x*-pos = 0.53, *y*-pos = 0.53 when using LSTM-RNN in the dataset CRT]. Decoding Z_DCV_ yielded the best performance of reconstructing the hand position using either LKF or LSTM-RNN (Friedman test, multiple comparison with Bonferroni correction, *p* < 0.05. See Tables 3, 4). The standard deviations (STDs) of the actual velocity and position in the dataset CRT are *X* = 0.24 and *Y* = 0.26 for velocity and *X* = 1.82, and *Y* = 1.76 for position, and those in the dataset SRT are *X* = 0.21 and *Y* = 0.20 for velocity and *X* = 1.66 and *Y* = 1.52 for position. For LKF, the decoding error is less than the STDs of the *X*- and *Y*-axes of the actual velocity by 5.7 and 4.2% on overage, respectively. Moreover, the decoding error is less than the STDs of the actual position by 72.1 and 69.1%. For LSTM-RNN, the decoding error is less than the STDs of the actual velocity by 5.7 and 4.6%, and the decoding error is less than those of the actual position by 72.3 and 70.0%.

**TABLE 3:** Correlation coefficients of the decoded velocity (datasets CRT and SRT).

LKF	Z_E–FR_	Z_PCA_	Z_FA_	Z_LDS_*	Z_LCV_	Z_DCV_
**CRT**						
*X*	0.64 ± 0.25	0.38 ± 0.30	0.72 ± 0.27	0.82 ± 0.21	0.73 ± 0.28	0.77 ± 0.33
*Y*	0.69 ± 0.18	0.62 ± 0.26	0.71 ± 0.20	0.84 ± 0.13	0.75 ± 0.18	0.84 ± 0.18
**SRT**						
*X*	0.58 ± 0.29	0.60 ± 0.40	0.66 ± 0.31	0.76 ± 0.24	0.66 ± 0.31	0.71 ± 0.31
*Y*	0.54 ± 0.29	0.51 ± 0.35	0.62 ± 0.31	0.59 ± 0.28	0.62 ± 0.32	0.64 ± 0.32

**LSTM-RNN**	**Z_E–FR_**	**Z_PCA_**	**Z_FA_**	**Z_LDS_**	**Z_LCV_**	**Z_DCV_**

**CRT**						
*X*	0.74 ± 0.26	0.70 ± 0.29	0.80 ± 0.20	0.82 ± 0.25	0.79 ± 0.32	0.80 ± 0.33
*Y*	0.81 ± 0.16	0.76 ± 0.22	0.83 ± 0.14	0.87 ± 0.14	0.87 ± 0.15	0.91 ± 0.11
**SRT**						
*X*	0.72 ± 0.31	0.74 ± 0.35	0.79 ± 0.27	0.78 ± 0.31	0.78 ± 0.28	0.79 ± 0.27
*Y*	0.69 ± 0.28	0.72 ± 0.31	0.73 ± 0.29	0.69 ± 0.36	0.75 ± 0.26	0.75 ± 0.28

Decoding error of Z_LDS_* corresponds to that of the NDF.

**TABLE 4:** Velocity and position decoding errors (datasets CRT/SRT).

LKF	Z_E–FR_	Z_PCA_	Z_FA_	Z_LDS_*	Z_LCV_	Z_DCV_
**Velocity (cm/s)**						
CRT	0.21 ± 0.04	0.28 ± 0.07	0.21 ± 0.04	0.16 ± 0.05	0.19 ± 0.04	0.13 ± 0.03
SRT	0.16 ± 0.05	0.17 ± 0.05	0.16 ± 0.04	0.15 ± 0.04	0.16 ± 0.05	0.15 ± 0.05
**Position (cm)**						
CRT	0.97 ± 0.36	1.20 ± 0.44	0.99 ± 0.34	0.91 ± 0.55	0.87 ± 0.32	0.57 ± 0.24
SRT	0.95 ± 0.60	1.01 ± 0.53	0.94 ± 0.43	0.90 ± 0.53	0.93 ± 0.48	0.83 ± 0.45

**LSTM-RNN**	**Z_E–FR_**	**Z_PCA_**	**Z_FA_**	**Z_LDS_**	**Z_LCV_**	**Z_DCV_**

**Velocity (cm/s)**						
CRT	0.18 ± 0.05	0.19 ± 0.05	0.16 ± 0.04	0.14 ± 0.05	0.13 ± 0.03	0.10 ± 0.02
SRT	0.14 ± 0.04	0.13 ± 0.04	0.12 ± 0.04	0.14 ± 0.06	0.12 ± 0.04	0.12 ± 0.05
**Position (cm)**						
CRT	1.15 ± 0.53	1.07 ± 0.43	0.92 ± 0.34	0.93 ± 0.50	0.65 ± 0.32	0.51 ± 0.22
SRT	0.86 ± 0.48	0.88 ± 0.52	0.78 ± 0.41	1.01 ± 0.60	0.82 ± 0.46	0.79 ± 0.46

Decoding error of Z_LDS_* corresponds to that of the NDF.

**FIGURE 8:**
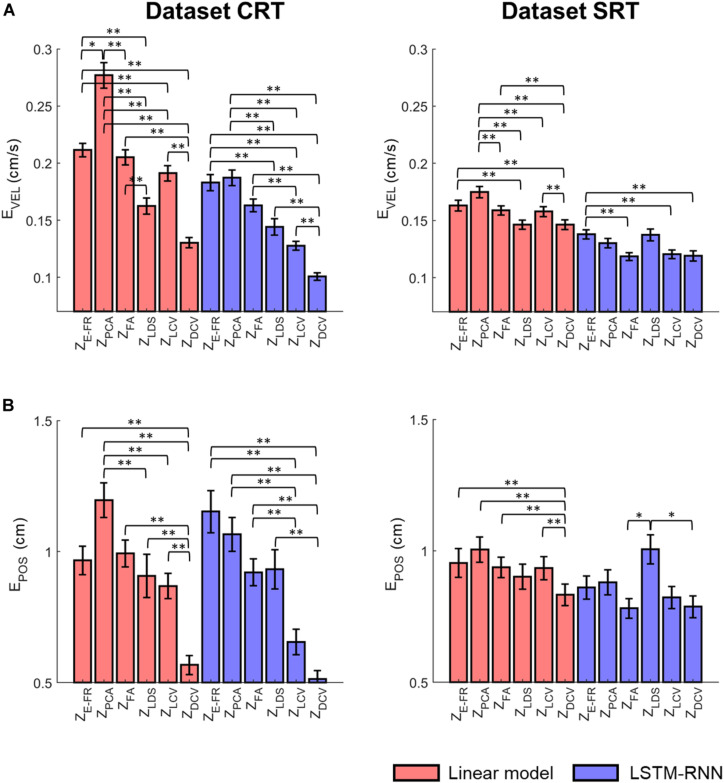
Comparison of the decoding error for the velocity between the neural representations for each decoder. The mean error of decoding the hand velocity **(A)** and reconstructing the hand position **(B)** from decoded velocity [from six different neural representations (i.e., Z_E–FR_, Z_PCA_, Z_FA_, Z_LDS_, Z_LCV_, and Z_DCV_)] (see the text for the descriptions of neural representations) using decoders [linear model (orange), and LSTM-RNN (purple)]. For linear model, LKF was used for Z_E–FR_, Z_PCA_, Z_FA_, Z_LCV_, and Z_DCV_, whereas NDF (linear filter) was used for Z_LDS_. The vertical lines indicate the standard error, and the asterisks denote the significantly different relationship [^∗^*p* < 0.05, ^∗∗^*p* < 0.01, Friedman test with the multiple comparisons (with Bonferroni correction)]. The left and right columns correspond to the dataset CRT and SRT, respectively.

Figure 8A depicts the comparison of the decoding error for the hand velocity across different neural representations and decoders. For the dataset CRT, the one-way Friedman test revealed the main effect of neural representation (Z_*FR*_, Z_PCA_, Z_FA_, Z_LDS_, Z_LCV_, and Z_DCV_) on the decoding error when using LKF (χ^2^ = 166.6, *p* < 0.01) or when using LSTM-RNN (χ^2^ = 128.1, *p* < 0.01). When using LKF, a *post hoc* multiple comparison test with Bonferroni correction showed lower decoding error with Z_DCV_ than other neural representations (*p*s < 0.01) except for Z_LDS_ (*p* = 0.25). When using LSTM-RNN, it also showed lower decoding error with Z_DCV_ than other neural representations (*p*s < 0.01). For the dataset SRT, the Friedman test revealed the main effect of neural representation on the decoding error when using LKF (χ^2^ = 75.8, *p* < 0.01) or when using LSTM-RNN (χ^2^ = 25.7, *p* < 0.01). When using LKF, the *post hoc* test showed lower decoding error with Z_DCV_ than other neural representations (*p*s < 0.01) except for Z_LDS_ (*p* ≅ 1). When using LSTM-RNN, it showed lower decoding error with Z_DCV_ than Z_E–FR_ (*p* < 0.01) only, while showing no difference between Z_DCV_ and other representations (*p*s > 0.05).

Figure 8B depicts the comparison of the error between true and reconstructed hand positions. For the dataset CRT, the Friedman test revealed the main effect of neural representation on the position error when using LKF (χ^2^ = 71.9, *p* < 0.01) or when using LSTM-RNN (χ^2^ = 80.7, *p* < 0.01). When using LKF, the *post hoc* test showed lower error with Z_DCV_ than neural representations (*p*s < 0.01). When using LSTM-RNN, it showed lower error with Z_DCV_ than other neural representations except for Z_LCV_ (*p* = 0.53). For the dataset SRT, the Friedman test revealed the main effect of the neural representation on the position error when using LKF (χ^2^ = 33.1, *p* < 0.01) or when using LSTM-RNN (χ^2^ = 13.6, *p* < 0.01). When using LKF, the *post hoc* test showed lower error with Z_DCV_ than other neural representations except for Z_LDS_ (*p* = 0.06). When using LSTM-RNN, it showed lower error with Z_DCV_ than Z_LDS_ (*p* < 0.01), whereas there was no difference between Z_DCV_ and others.

**FIGURE 9:**
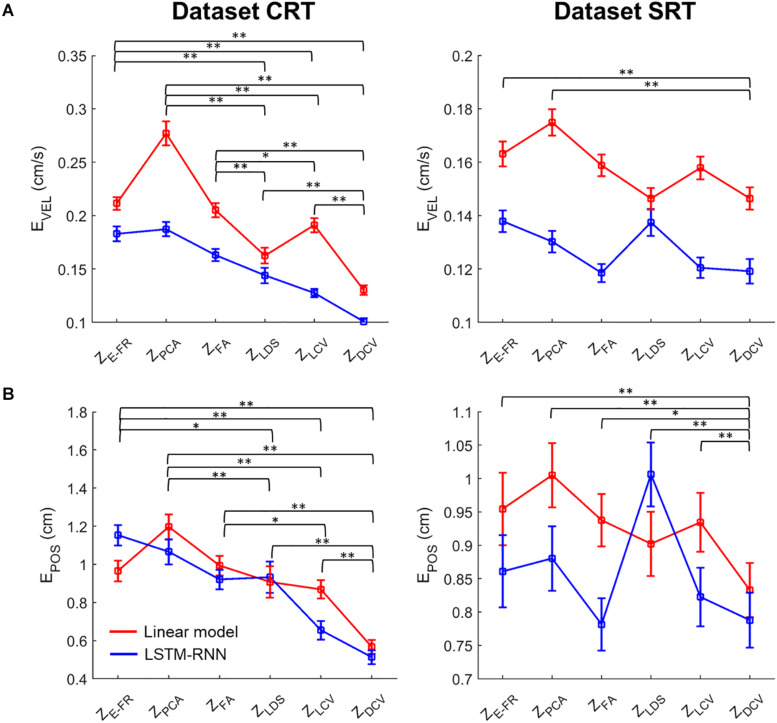
Comparison of the decoding error for the velocity and reconstructed position between neural representations for all decoders. The mean error (open squares) of decoding the hand **(A)** velocity and **(B)** position from the six different neural representations (i.e., Z_E–FR_, Z_PCA_, Z_FA_, Z_LDS_, Z_LCV_, and Z_DCV_) (see the text for descriptions of neural representations) using decoders [linear model (red), and LSTM-RNN (blue)]. For linear model, LKF was used for Z_E–FR_, Z_PCA_, Z_FA_, Z_LCV_, and Z_DCV_, whereas NDF (linear filter) was used for Z_LDS_. The vertical lines indicate the standard error, and the asterisks denote the significantly different relationship [^∗^*p* < 0.05, ^∗∗^*p* < 0.01, a two-way Friedman test with the multiple comparisons (with Bonferroni correction)]. The left and right columns correspond to the dataset CRT and SRT, respectively.

Moreover, we evaluated the possible interaction effects of neural representations and decoder types using a two-way Friedman test (Figure 9). For the dataset CRT, the two-way Friedman test revealed the main effects of decoder [χ^2^(1) = 116.9, *p* < 0.01] and neural representation [χ^2^(2) = 261.9, *p* < 0.01] on the velocity decoding error (Figure 9A). The *post hoc* test with Bonferroni correction showed lower error using LSTM-RNN than using LKF for all neural representations (*p* < 0.01). For all decoders, the decoding error of velocity with Z_DCV_ was smaller than any other neural representations (*p*s < 0.01). For the dataset SRT, the two-way Friedman test revealed the main effect of decoder [χ^2^(1) = 175.4, *p* < 0.01] and neural representation [χ^2^(2) = 59.0, *p* < 0.01]. The *post hoc* test showed lower error using LSTM-RNN than using LKF (*p* < 0.01). For all decoders, the decoding error of velocity with Z_DCV_ was smaller than Z_E–FR_ and Z_PCA_ (*p*s < 0.01).

Figure 9B depicts the same two-way statistical analysis on the error between true and reconstructed hand positions. For the dataset CRT, the two-way Friedman test revealed the main effects of decoder [χ^2^(1) = 4.4, *p* < 0.05] and neural representation [χ^2^(2) = 143.1, *p* < 0.01] on the position error. The *post hoc* test showed no difference between decoders (*p* = 0.3). For all decoders, the position error with Z_DCV_ was smaller than any other neural representations (*p*s < 0.01). For the dataset SRT, it showed the main effects of decoder [χ^2^(1) = 14.3, *p* < 0.01] and neural representation [χ^2^(2) = 28.2, *p* < 0.01]. The *post hoc* test showed no difference between decoders (*p* = 0.1). For all decoders, the position error with Z_DCV_ was smaller than any other neural representations (*p*s < 0.05).

## Discussion

In this study, we proposed a method to identify low-dimensional representations of M1 neuronal FR activities using canonical correlation analyses. Furthermore, we applied those canonical variables to the decoding models to predict the arm movements of non-human primates and compared the effect of the neural representations in terms of decoding performance. As expected, we confirmed that the canonical variables found by DCCA were well tuned to the hand velocity. Decoding arm movement information using canonical variables estimated by DCCA resulted in a superior performance to either cases using LCCA-estimated canonical variables or using the other neural representations regardless of the decoder type, i.e., LKF or LSTM-RNN. In particular, the performance of LKF was significantly more improved using DCCA than decoding FRs using LSTM-RNN. These findings suggest that we can design a simple linear decoder (LKF) with DCCA while achieving performance as good as using relatively complex DNNs.

The improvement of decoding M1 activities using LCCA or DCCA may be partly because canonical variables found by them showed superior tuning to velocity over the other neural representations, including individual neuronal FRs (Figure 3). Therefore, the LKF, drawing heavily on the quality of observation models, can benefit from the extracted canonical variables even when LCCA greatly reduced the number of neural variables. Particularly, DCCA-estimated neural canonical variables showed better tuning indices (*r*^2^) than LCCA-estimated neural canonical variables, which subsequently led to a better decoding performance of DCCA. Meanwhile, training error that directly reflects the learning quality of the decoding model revealed superior over the other neural representations along with *r*^2^. This finding indicates that non-linear projections may be more suitable to extract joint canonical variables between high-dimensional neural activities and low-dimensional kinematic parameters. However, DCCA cannot provide direct links between canonical variables and individual neurons, which LCCA can do.

Besides better characteristics of the canonical variables, there could be another reason why DCCA improved decoding using LKF while the other neural representations did not. PCA is known to have difficulty distinguishing between changes in the underlying neural state, which becomes limitations to decoding kinematic information from noisy firing activity (Yu et al., 2009). Although FA also is a useful frame to extract independent and shared variability across neurons, it follows the assumption that the noise variance of an individual neuron is fixed over time (Yu et al., 2009). Above all, since these approaches (including LDS) aim to extract the latent states of population activity without kinematic information, it is difficult to extract elaborate components related to complex movement. This could be a reasonable reason why DCCA yielded a better performance on decoding models than the neural representations via unsupervised learning methods.

As for decoding methods, a DNN, represented by LSTM-RNN here, efficiently decoded neuronal population firing patterns because it can effectively process neuronal temporal dynamics through memory cells in a relatively concise network architecture. Furthermore, a state-space model, such as LKF, shows an advantage of representing temporal dynamics of kinematics in its system model, but its first-order linear system model may not be sufficient to elucidate the kinematic dynamics of arm movements. In addition, a direct decoding model, such as LSTM-RNN, can be free from any statistical assumption on data, which is often necessary in a generative model, such as LKF. Our results showing the superior performance of LSTM-RNN over LKF are in line with those of previous reports (e.g., Ahmadi et al., 2019).

In addition to direct mapping to velocity through the decoders, a more straightforward linear mapping could be taken into account; for example, we can simply reconstruct velocity from the canonical kinematic representations (X_LCV_ or X_DCV_), which were estimated from the corresponding neural representations (Z_LCV_ or Z_DCV_). To test whether how this simple mapping worked, we attempted to reconstruct velocity only through LCCA and DCCA without explicit decoders as follows. First, we estimated X_LCV_ (or X_DCV_) from Z_LCV_ (or Z_DCV_) by linear regression such as

(18) $$X_{\text{k}}=\alpha_{0}+\alpha_{1}Z_{\text{k}}+e$$

where *X*_*k*_ and *Z*_*k*_ represent the *k*-th canonical variable, respectively, *e* represents residual error and *α*_0_ and *α*_1_ are canonical coefficients. Second, we reconstructed velocity from the estimated X_LCV_ (or X_DCV_) during testing. For LCCA, the reconstruction of velocity was straightforward simply by inverting linear mapping between X_LCV_ and velocity. For DCCA, velocity was reconstructed by the inverse of activation function (here, a logit function) and the linear model between the layers, which was expressed as:

(19) $$-\log\left({\left(\beta_{l,l-1}XW_{l}^{-1}-1\right)}^{-1}\right)$$

where β_*l,l*__–__1_ represents coefficients between the outputs of layer *l* and *l−*1, and *W*_*l*_ is a matrix of the weight in *l*-th layer. We observed that the reconstructed velocity with this procedure exhibited lower performance than directly decoding Z (Z_LCV_ or Z_DCV_) into velocity using LKF by 9.9% on average (11.4% for LCCA, and 8.3% for DCCA). Apparently, this analysis verified that direct reconstruction of velocity through mappings built by CCA was poorer than those from the proposed decoding methods to predict velocity from neural representations using LKF or LSTM-RNN.

We can expect that the dimensionality of neuronal populations will increase further as the neurotechnology of large-scale neuronal recordings advances in the near future. Such a development will raise an issue of how efficiently we should design a decoder for intracortical BMIs. Our results suggest that DCCA, along with other dimensionality reduction techniques, can provide advantages to construct a compact but informative feature space for effective decoding. Unlike unsupervised dimensionality reduction techniques without kinematic information, DCCA can find a low-dimensional space to maximize correlations with target kinematic parameters, increasing a chance to improve predicting kinematic parameters such as velocity from neural activities. It has been well known that decoding velocity information of a prosthetic device from neural activity can be useful for BMIs in clinical environments (Kim et al., 2008; Wodlinger et al., 2015). Therefore, we suggest that our proposal method can be preferred if one considers the efficiency and performance of BMIs.

Although this study shows the feasibility of the improvement of decoding for BMIs using the proposed method, we have not validated it in an online BMI control paradigm, which should be conducted in future work. When applying the current decoding method to online BMIs in humans with tetraplegia, where the kinematic information of limbs is not available, we should consider how to extract kinematics of a target prosthetic device. To address this issue, many previous human BMI studies employed a training paradigm in which participants imagined limb movements following the instructed motion of an object shown on the screen. Then, a decoding algorithm could be built by associating M1 activities elicited during movement imagination with the kinematics of the object (Hochberg et al., 2006; Kim et al., 2008; Aflalo et al., 2015; Jarosiewicz et al., 2015; Wodlinger et al., 2015). Although there could exist a substantial gap between the true kinematics and the output of the decoding algorithm initially built in this way, the BMI performance could be further increased by repeatedly updating the same decoding algorithm through “closed-loop” training. Importantly, most decoding algorithms used in human BMIs have been initially developed in the preliminary non-human primate studies. Therefore, we believe that our decoding algorithm based on deep CCA in non-human primates can benefit human BMIs in a similar fashion.

## Data Availability Statement

Publicly available datasets were analyzed in this study. This data can be found here: http://crcns.org/data-sets/movements/dream and http://crcns.org/data-sets/motor-cortex/pmd-1/about-pmd-1.

## Author Contributions

M-KK and S-PK conceived the research. M-KK conducted the analysis and wrote the manuscript. J-WS and S-PK provided edited the manuscript. All authors contributed to the article and approved the submitted version.

## Conflict of Interest

The authors declare that the research was conducted in the absence of any commercial or financial relationships that could be construed as a potential conflict of interest.

## References

[B1] Aflalo T. Kellis S. Klaes C. Lee B. Shi Y. Pejsa K. (2015). Decoding motor imagery from the posterior parietal cortex of a tetraplegic human. *Science* 348 906–910. 10.1126/science.aaa5417 25999506 PMC4896830

[B2] Aggarwal V. Acharya S. Tenore F. Shin H. C. Etienne-Cummings R. Schieber M. H. (2008). Asynchronous decoding of dexterous finger movements using M1 neurons. *IEEE Trans. Neural. Syst. Rehabil. Eng.* 16 3–14. 10.1109/TNSRE.2007.916289 18303800 PMC2780566

[B3] Ahmadi N. Constandinou T. G. Bouganis C.-S. (2019). “Decoding Hand Kinematics from Local Field Potentials Using Long Short-Term Memory (LSTM) Network,” in *2019 9th International IEEE/EMBS Conference on Neural Engineering (NER)* (San Francisco, CA: Cornell University), 415–419. 10.1109/NER.2019.8717045

[B4] Ames K. C. Ryu S. I. Shenoy K. V. (2014). Neural dynamics of reaching following incorrect or absent motor preparation. *Neuron* 81 438–451. 10.1016/j.neuron.2013.11.003 24462104 PMC3936035

[B5] Anderson T. W. (1984). *An Introduction to Multivariate Statistical Analysis*, 2nd Edn New Jersey: John Wiley and Sons.

[B6] Andrew G. Aroral R. Bilmes J. Livescu K. (2013). “Deep Canonical Correlation Analysis,” in *Proceedings of the 30th International Conference on Machine Learning* (Atlanta: University of Washington), 1247–1255.

[B7] Chapin J. K. Moxon K. A. Markowitz R. S. Nicolelis M. A. L. (1999). Real-time control of a robot arm using simultaneously recorded neurons in the motor cortex. *Nat. Neurosci.* 2 664–670. 10.1038/10223 10404201

[B8] Chhatbar P. Y. Francis J. T. (2013). Towards a Naturalistic Brain-Machine Interface: Hybrid Torque and Position Control Allows Generalization to Novel Dynamics. *PLoS One.* 8:e52286. 10.1371/journal.pone.0052286 23359212 PMC3554733

[B9] Cunningham J. P. Yu B. M. (2014). Dimensionality reduction for large-scale neural recordings. *Nat. Neurosci.* 17 1500–1509. 10.1038/nn.3776 25151264 PMC4433019

[B10] Dethier J. Nuyujukian P. Ryu S. I. Shenoy K. V. Boahen K. (2013). Design and validation of a real-time spiking-neural-network decoder for brain-machine interfaces. *J. Neural. Eng.* 10:036008 10.1088/1741-2560/10/3/036008 PMC3674827 23574919

[B11] Fagg A. H. Ojakangas G. W. Miller L. E. Hatsopoulos N. G. (2009). Kinetic trajectory decoding using motor cortical ensembles. *IEEE Trans. Neural. Syst. Rehabil. Eng.* 17 487–496. 10.1109/TNSRE.2009.2029313 19666343

[B12] Flament D. Hore J. (1988). Relations of motor cortex neural discharge to kinematics of passive and active elbow movements in the monkey. *J. Neurophysiol.* 60 1268–1284. 10.1152/jn.1988.60.4.1268 3193157

[B13] Flint R. D. Lindberg E. W. Jordan L. R. Miller L. E. Slutzky M. W. (2012). Accurate decoding of reaching movements from field potentials in the absence of spikes. *J. Neural. Eng.* 9 46006 10.1088/1741-2560/9/4/046006 PMC3429374 22733013

[B14] Friman O. Borga M. Lundberg P. Knutsson H. (2007). “Canonical Correlation Analysis: in Applied Multivariate Statistical Analysis,” in *Applied Multivariate Statistical Analysis* (Berlin: Springer), 321–330. 10.1007/978-3-540-72244-1_14

[B15] Gao Y. Black M. J. Bienenstock E. Shoham S. Donoghue J. P. (2001). “Probabilistic Inference of Hand Motion from Neural Activity in Motor Cortex,” in *Proceedings of the 14th International Conference on Neural Information Processing Systems: Natural and Synthetic* (Canada: MIT Press), 213–220. 10.7551/mitpress/1120.003.0032

[B16] Georgopoulos A. P. Kalaska J. F. Caminiti R. Massey J. T. (1982). On the relations between the direction of two-dimensional arm movements and cell discharge in primate motor cortex. *J. Neurosci.* 2 1527–1537. 10.1523/jneurosci.02-11-01527.1982 7143039 PMC6564361

[B17] Georgopoulos A. P. Kettner R. E. Schwartz A. B. (1988). Primate motor cortex and free arm movements to visual targets in three-dimensional space. *II. Coding of the direction of movement by a neuronal population*. *J. Neurosci.* 8 2928–2937. 10.1523/jneurosci.08-08-02928.1988 3411362 PMC6569382

[B18] Georgopoulos A. P. Schwartz A. B. Kettner R. E. (1986). Neuronal Population Coding of Movement Direction. *Science* 233 1416–1419. 10.1126/science.3749885 3749885

[B19] Gilja V. Nuyujukian P. Chestek C. A. Cunningham J. P. Yu B. M. Fan J. M. (2012). A high-performance neural prosthesis enabled by control algorithm design. *Nat. Neurosci.* 15 1752–1757. 10.1038/nn.3265 23160043 PMC3638087

[B20] Golub M. D. Yu B. M. Schwartz A. B. Chase S. M. (2014). Motor cortical control of movement speed with implications for brain-machine interface control. *J. Neurophysiol.* 112 411–429. 10.1152/jn.00391.2013 24717350 PMC4064402

[B21] Hochberg L. R. Serruya M. D. Friehs G. M. Mukand J. A. Saleh M. Caplan A. H. (2006). Neuronal ensemble control of prosthetic devices by a human with tetraplegia. *Nature* 442 164–171. 10.1038/nature04970 16838014

[B22] Hotelling H. (1936). Relations between two sets of variates. *Biometrika* 28 321–377. 10.2307/2333955

[B23] Humphrey D. R. (1972). Relating motor cortex spike trains to measures of motor performance. *Brain Res.* 40 7–18. 10.1016/0006-8993(72)9009990096 4624492

[B24] Humphrey D. R. Corrie W. S. (1978). Properties of pyramidal tract neuron system within a functionally defined subregion of primate motor cortex. *J. Neurophysiol.* 41 216–243. 10.1152/jn.1978.41.1.216 413887

[B25] Jarosiewicz B. Sarma A. A. Bacher D. Masse N. Y. Simeral J. D. Sorice B. (2015). Virtual typing by people with tetraplegia using a self-calibrating intracortical brain-computer interface. *Sci. Transl. Med.* 7:313ra179. 10.1126/scitranslmed.aac7328 26560357 PMC4765319

[B26] Kao J. C. Nuyujukian P. Ryu S. I. Churchland M. M. Cunningham J. P. Shenoy K. V. (2015). Single-trial dynamics of motor cortex and their applications to brain-machine interfaces. *Nat. Commun.* 6 1–12. 10.1038/ncomms8759 26220660 PMC4532790

[B27] Kao J. C. Nuyujukian P. Stavisky S. Ryu S. I. Ganguli S. Shenoy K. V. (2013). Investigating the role of firing-rate normalization and dimensionality reduction in brain-machine interface robustness. *Proc. Annu. Int. Conf. IEEE Engin. Med. Biol. Soc.* 2013 293–298. 10.1109/EMBC.2013.6609495 24109682

[B28] Kaufman M. T. Churchland M. M. Ryu S. I. Shenoy K. V. (2014). Cortical activity in the null space: Permitting preparation without movement. *Nat. Neurosci.* 17 440–448. 10.1038/nn.3643 24487233 PMC3955357

[B29] Kim K. H. Kim S. S. Kim S. J. (2006). Superiority of nonlinear mapping in decoding multiple single-unit neuronal spike trains: A simulation study. *J. Neurosci. Methods* 150 202–211. 10.1016/j.jneumeth.2005.06.015 16099513

[B30] Kim S. P. Sanchez J. C. Erdogmus D. Rao Y. N. Wessberg J. Principe J. C. (2003). Divide-and-conquer approach for brain machine interfaces: Nonlinear mixture of competitive linear models. *Neural. Networks* 16 865–871. 10.1016/S0893-6080(03)00108104 12850045

[B31] Kim S. P. Simeral J. D. Hochberg L. R. Donoghue J. P. Black M. J. (2008). Neural control of computer cursor velocity by decoding motor cortical spiking activity in humans with tetraplegia. *J. Neural. Eng.* 5 455–476. 10.1088/1741-2560/5/4/010 19015583 PMC2911243

[B32] Lawlor P. N. Perich M. G. Miller L. E. Kording K. P. (2018). Linear-nonlinear-time-warp-poisson models of neural activity. *J. Comput. Neurosci.* 45 173–191. 10.1007/s10827-018-0696696 30294750 PMC6409107

[B33] Liu W. Qiu J.-L. Zheng W.-L. Lu B.-L. (2019). *Multimodal emotion recognition using deep canonical correlation analysis.* arXiv Preprint arXiv:1908.05349.

[B34] Marblestone A. H. Zamft B. M. Maguire Y. G. Shapiro M. G. Cybulski T. R. Glaser J. I. (2013). Physical principles for scalable neural recording. *Front. Comput. Neurosci.* 7 1–34. 10.3389/fncom.2013.00137 24187539 PMC3807567

[B35] Moran D. W. Schwartz A. B. (1999a). Motor cortical activity during drawing movements: Population representation during spiral tracing. *J. Neurophysiol.* 82 2693–2704. 10.1152/jn.1999.82.5.2693 10561438

[B36] Moran D. W. Schwartz A. B. (1999b). Motor cortical representation of speed and direction during reaching. *J. Neurophysiol.* 82 2676–2692. 10.1152/jn.1999.82.5.2676 10561437

[B37] Paninski L. Fellows M. R. Hatsopoulos N. G. Donoghue J. P. (2003). Spatiotemporal Tuning of Motor Cortical Neurons for Hand Position and Velocity. *J. Neurophysiol.* 91 515–532. 10.1152/jn.00587.2002 13679402

[B38] Perich M. G. Lawlor P. N. Kording K. P. Miller L. E. (2018). *Extracellular neural recordings from macaque primary and dorsal premotor motor cortex during a sequential reaching task.* Available online at: crcns.org.http://dx.10.6080/K0FT8J72 (accessed May, 2019).

[B39] Qiu J. L. Liu W. Lu B. L. (2018). “Multi-view emotion recognition using deep canonical correlation analysis,” in *25th International Conference, ICONIP 2018.* (Berlin: Springer).

[B40] Rao Y. N. Kim S. P. Sanchez J. C. Erdogmus D. Principe J. C. Carmena J. M. (2005). “Learning mappings in brain machine interfaces with echo state networks. in Proceedings. (ICASSP ’05),” in *IEEE International Conference on Acoustics, Speech, and Signal Processing* (New Jersey: IEEE), 233–236. 10.1109/ICASSP.2005.1416283

[B41] Rasmussen R. G. Schwartz A. Chase S. M. (2017). Dynamic range adaptation in primary motor cortical populations. *Elife* 6:e21409. 10.7554/eLife.21409 28417848 PMC5395298

[B42] Sanchez J. C. Erdogmus D. Rao Y. Principe J. C. Nicolelis M. Wessberg J. (2003). “Learning the contributions of the motor, premotor, and posterior parietal cortices for hand trajectory reconstruction in a brain machine interface,” in *First International IEEE EMBS Conference on Neural Engineering, 2003. Conference Proceedings* (New Jersey: IEEE), 59–62. 10.1109/CNE.2003.1196755

[B43] Santhanam G. Yu B. M. Gilja V. Ryu S. I. Afshar A. Sahani M. (2009). Factor-analysis methods for higher-performance neural prostheses. *J. Neurophysiol.* 102 1315–1330. 10.1152/jn.00097.2009 19297518 PMC2724333

[B44] Schwartz A. B. (2007). Useful signals from motor cortex. *J. Physiol.* 579 581–601. 10.1113/jphysiol.2006.126698 17255162 PMC2151362

[B45] Schwartz A. B. Kettner R. E. Georgopoulos A. P. (1988). Primate motor cortex and free arm movements to visual targets in three- dimensional space. *I. Relations between single cell discharge and direction of movement*. *J. Neurosci.* 8 2913–2927. 10.1523/JNEUROSCI.08-08-02913.1988 3411361 PMC6569414

[B46] Schwartz A. B. Moran D. W. (1999). Motor cortical representation of speed and direction during reaching. *J. Neurophysiol.* 82 2676–2692. 10.1152/jn.1999.82.5.2676 10561437

[B47] Schwartz A. B. Moran D. W. (2000). Arm trajectory and representation of movement processing in motor cortical activity. *Eur. J. Neurosci.* 12 1851–1856. 10.1046/j.1460-9568.2000.00097.x 10886326

[B48] Sergio L. E. Hamel-Pâquet C. Kalaska J. F. (2005). Motor cortex neural correlates of output kinematics and kinetics during isometric-force and arm-reaching tasks. *J. Neurophysiol.* 94 2353–2378. 10.1152/jn.00989.2004 15888522

[B49] Serruya M. D. Hatsopoulos N. G. Paninski L. Donoghue M. R. F. J. P. (2002). Instant neural control of a movement signal. *Nature* 416 141–142. 10.1038/416141a 11894084

[B50] Shanechi M. M. Orsborn A. Moorman H. Gowda S. Carmena J. M. (2014). “High-performance brain-machine interface enabled by an adaptive optimal feedback-controlled point process decoder,” in *2014 36th Annual International Conference of the IEEE Engineering in Medicine and Biology Society* (New Jersey: IEEE), 6493–6496. 10.1109/EMBC.2014.6945115 25571483

[B51] Shanechi M. M. Orsborn A. L. Carmena J. M. (2016). Robust Brain-Machine Interface Design Using Optimal Feedback Control Modeling and Adaptive Point Process Filtering. *PLoS Comput. Biol.* 12:e1004730. 10.1371/journal.pcbi.1004730 27035820 PMC4818102

[B52] Shenoy K. V. Sahani M. Churchland M. M. (2013). Cortical control of arm movements: A dynamical systems perspective. *Annu. Rev. Neurosci.* 36 337–359. 10.1146/annurev-neuro-062111150509 23725001

[B53] Suminski A. J. Tkach D. C. Fagg A. H. Hatsopoulos N. G. (2010). Incorporating feedback from multiple sensory modalities enhances brain-machine interface control. *J. Neurosci.* 30 16777–16787. 10.1038/mp.2011.182 21159949 PMC3046069

[B54] Sussillo D. Nuyujukian P. Fan J. M. Kao J. C. Stavisky S. D. Ryu S. (2012). A recurrent neural network for closed-loop intracortical brain-machine interface decoders. *J. Neural. Eng.* 9 026027 10.1088/1741-2560/9/2/026027 PMC3638090 22427488

[B55] Van Hemmen J. L. Schwartz A. B. (2008). Population vector code: a geometric universal as actuator. *Biol. Cybern.* 98 509–518. 10.1007/s00422-008-0215213 18491163

[B56] Vargas-Irwin C. E. Shakhnarovich G. Yadollahpour P. Mislow J. M. K. Black M. J. Donoghue J. P. (2010). Decoding complete reach and grasp actions from local primary motor cortex populations. *J. Neurosci.* 30 9659–9669. 10.1523/JNEUROSCI.5443-09.2010 20660249 PMC2921895

[B57] Vaskov A. K. Irwin Z. T. Nason S. R. Vu P. P. Nu C. S. Bullard A. J. (2018). Cortical Decoding of Individual Finger Group Motions Using ReFIT Kalman Filter. *Front. Neurosci.* 12:751. 10.3389/fnins.2018.00751 30455621 PMC6231049

[B58] Vincent P. Larochelle H. Bengio Y. Manzagol P.-A. (2008). “Extracting and composing robust features with denoising autoencoders,” in *Proceeding ICML ’08 Proceedings of the 25th international conference on Machine learning* (New York: ACM Press), 1096–1103. 10.1145/1390156.1390294

[B59] Vu H. Koo B. Choi S. (2016). “Frequency detection for SSVEP-based BCI using deep canonical correlation analysis,” in *2016 IEEE International Conference on Systems, Man, and Cybernetics* (Tokyo: SMC), 001983–001987.

[B60] Wang W. Arora R. Livescu K. Bilmes J. (2015). *MATLAB package for Deep Canonically Correlated Autoencoders (DCCAE).* Available online at: https://ttic.uchicago.edu/∼wwang5/dccae.html. (accessed January 2019).

[B61] Wang W. Chan S. S. Heldman D. A. Moran D. W. (2007). Motor Cortical Representation of Position and Velocity During Reaching. *J. Neurophysiol.* 97 4258–4270. 10.1152/jn.01180.2006 17392416

[B62] Wang Y. Kim S. P. Principe J. C. (2005). “Comparison of TDNN training algorithms in brain machine interfaces,” in *Proceedings. 2005 IEEE International Joint Conference on Neural Networks* (New Jersey: IEEE), 2459–2462. 10.1109/IJCNN.2005.1556288

[B63] Wang Y. Paiva A. R. C. Príncipe J. C. Sanchez J. C. (2009). Sequential Monte Carlo Point-Process Estimation of Kinematics from Neural Spiking Activity for Brain-Machine Interfaces. *Neural. Comput.* 21 2894–2930. 10.1162/neco.2009.01-08699 19548797

[B64] Wessberg J. Stambaugh C. R. Kralik J. D. Beck P. D. Laubach M. Chapin J. K. (2000). Real-time prediction of hand trajectory by ensembles of cortical neurons in primates. *Nature* 408 361–365. 10.1038/35042582 11099043

[B65] Willett F. R. Suminski A. J. Fagg A. H. Hatsopoulos N. G. (2013). Improving brain-machine interface performance by decoding intended future movements. *J. Neural. Eng.* 10 026011 10.1088/1741-2560/10/2/026011 PMC4019387 23428966

[B66] Wodlinger B. Downey J. E. Tyler-Kabara E. C. Schwartz A. B. Boninger M. L. Collinger J. L. (2015). Ten-dimensional anthropomorphic arm control in a human brain-machine interface: Difficulties, solutions, and limitations. *J. Neural. Eng.* 12 1–17. 10.1088/1741-2560/12/1/016011 25514320

[B67] Wu W. Black M. J. Mumford D. Gao Y. Bienenstock E. Donoghue J. P. (2004). Modeling and Decoding Motor Cortical Activity using a Switching Kalman Filter. *IEEE Trans. Biomed. Eng.* 51 933–942. 10.1109/TBME.2004.826666 15188861

[B68] Wu W. Gao Y. Bienenstock E. Donoghue J. P. Black M. J. (2006). Bayesian population decoding of motor cortical activity using a Kalman filter. *Neural. Comput.* 18 80–118. 10.1162/089976606774841585 16354382

[B69] Xu K. Wang Y. Zhang S. Zhao T. Wang Y. Chen W. (2011). “Comparisons between linear and nonlinear methods for decoding motor cortical activities of monkey,” in *2011 Annual International Conference of the IEEE Engineering in Medicine and Biology Society* (New Jersey: IEEE), 4207–4210. 10.1109/IEMBS.2011.6091044 22255267

[B70] Yu B. M. Cunningham J. P. Santhanam G. Ryu S. I. Shenoy K. V. Sahani M. (2009). Gaussian-process factor analysis for low-dimensional single-trial analysis of neural population activity. *J. Neurophysiol.* 102 614–635. 10.1152/jn.90941.2008 19357332 PMC2712272

